# Use of virtual reality for epidural placement in an adolescent with ischemic priapism

**DOI:** 10.1002/pne2.12021

**Published:** 2020-04-30

**Authors:** Zvonimir Bebic, James Joseph Thomas

**Affiliations:** ^1^ Section of Pediatric Anesthesiology Department of Anesthesiology Children’s Hospital Colorado University of Colorado School of Medicine Aurora CO USA

**Keywords:** acute pain, chronic pain, pediatric, regional anesthesia, sickle cell disease, virtual reality

## Abstract

In children with chronic pain conditions, the acute pain and anxiety induced by routine procedures such as dressing changes, phlebotomy, and lumbar punctures may be amplified compared to that experienced by healthy children. However, sedatives and opiates may be contraindicated if respiratory depression is a concern. In this case report, we describe a 17‐year‐old male with ischemic priapism secondary to sickle cell disease in whom we used virtual reality immersion as a distraction method during epidural catheter placement. No sedation or analgesia was needed, and the patient reported no pain or distress.

## 1 INTRODUCTION

Common procedures such as dressing changes, phlebotomy, and lumbar punctures can produce pain and anxiety for children that can lead to maladaptive behaviors such as feeding difficulties, withdrawal, enuresis, and apathy.^1^ In children with a history of repeated needle procedures, such as those with sickle cell disease (SCD), increased fearfulness, distrust of adults, and reduced sense of control over their health may develop.^2^ Thus, the associated social, psychological, and biologic factors benefit from a multimodal and multidisciplinary approach of which virtual reality (VR) can be a component.^3^, ^4^, ^5^ VR is an immersive technology that has been shown to decrease pain, anxiety, and the need for sedation during needle‐based procedures.^6^, ^7^ We describe a case of epidural placement in an adolescent male with ischemic priapism secondary to SCD in which the use of family education and VR obviated the need for sedation or analgesia.

## 2 CASE DESCRIPTION

A 17‐year‐old, 75‐kg African American male with SCD was admitted to a quaternary children's hospital with painful ischemic priapism. After the patient was hydrated, the Pediatric Urology service aspirated and irrigated the corpora with 400 mcg of phenylephrine. The procedure was successful initially, but the patient experienced a return of priapism. He was administered opiates for analgesia that led to respiratory depression and hypoxia. The acute pain service was consulted to place a lumbar epidural for analgesia and treatment of low‐flow priapism.^8^ The patient and mother were anxious regarding pain associated with epidural placement. However, owing to the previous hypoxia, they were reluctant to have additional sedation.

**Figure 1:**
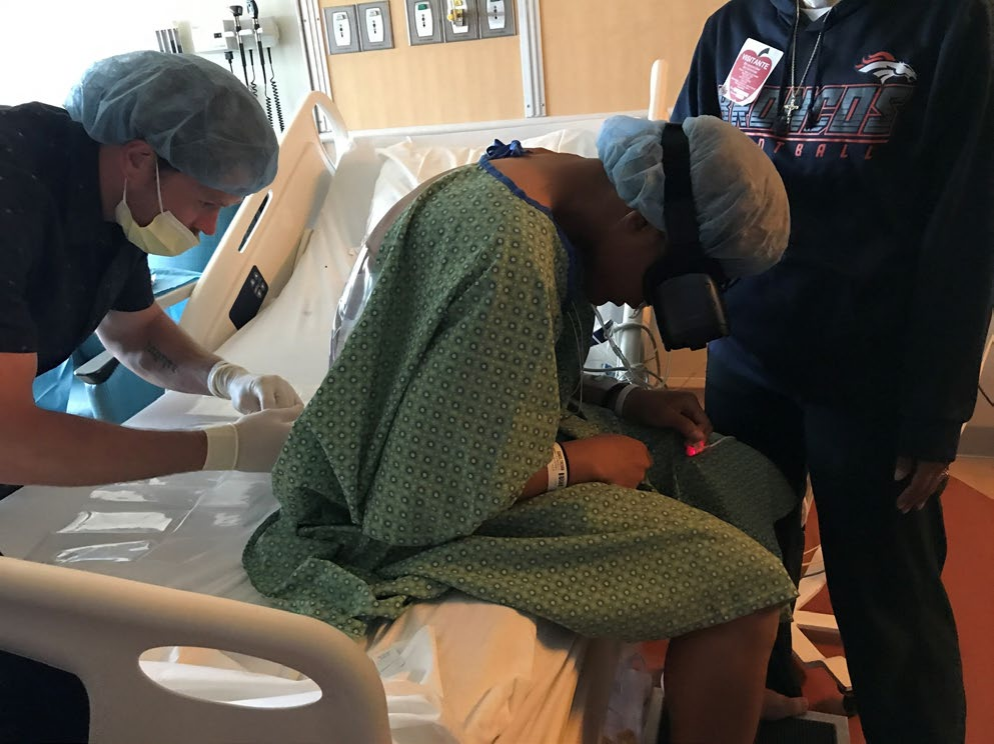
Patient positioned with VR headset

**Figure 2:**
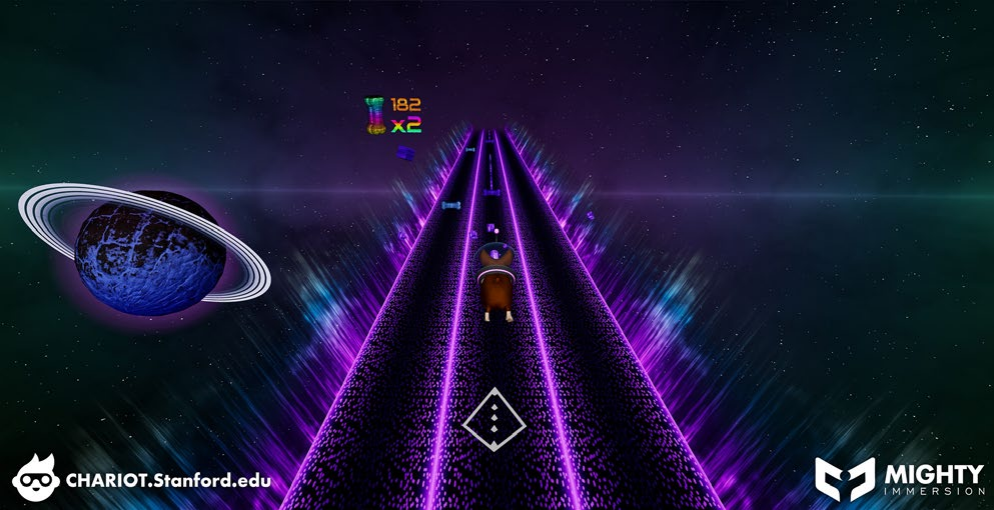
Space pups courtesy of mighty immersion

After obtaining patient and parental consent, the pain service proposed the use of immersive VR instead of sedation. Per our institution's standard, a child life specialist (CLS) provided counseling and education to both the patient and mother after which the patient was positioned upright, and the device (Mirage, Lenovo, Quarry Bay, Hong Kong) was placed over his head (Figure 1). Following a brief orientation, the patient was allowed to immerse himself in the program (Space Pups, courtesy of Mighty Immersion, Figure 2). The patient's back was prepped and draped in sterile fashion with chlorhexidine 3%. Lidocaine 1% was administered subcutaneously at approximately the L4‐5 interspinous level and an 18‐gauge Tuohy needle inserted. The patient initially made a brief moaning noise but tolerated the procedure well. As the epidural catheter was placed and tested successfully, the patient's vital signs remained stable without sedation. Parental presence was maintained throughout and the CLS was present to offer reassurance. After the procedure was completed, the patient denied anxiety or discomfort, and when questioned about his initial reaction, he was unable to recall. The mother was elated with her son's VR experience.

## 3 DISCUSSION

This case report demonstrates a novel use of VR for distraction and medication reduction in a patient with a chronic pain condition. We found immersive VR to be beneficial in our patient during this painful and anxiety‐provoking procedure as it eliminated the need for further sedation while providing a nonopioid modality of analgesia. Acute pain crises from SCD are often incompletely managed, and patients are often treated with opioids with minimal success.^9^ Immersive VR has been shown to decrease pain intensity and pain descriptors for pediatric patients in acute pain crisis from SCD.^10^ To date, there are no reports of the use of VR for placement of an epidural catheter in an adolescent patient but our experience is consistent with studies that endorse its use to reduce pain and anxiety in children during invasive procedures.^6^ Given its safety and ease of use, further investigation is needed to validate the efficacy of VR for invasive procedures in pediatric patients.

## CONFLICTS OF INTEREST

Consent for publication and use of pictures was obtained from the patient and mother. The authors have no conflicts of interest to disclose.
